# The role of women's traditional gender beliefs in depression, intimate partner violence and stress: insights from a Spanish abbreviated multicultural measure

**DOI:** 10.1186/s12905-021-01572-2

**Published:** 2022-01-22

**Authors:** Montse Rovira, Leonor Lega, Carlos Suso-Ribera, Izaskun Orue

**Affiliations:** 1 University of Deusto, Bilbao, Spain; 2 Saint Peter’s University, Jersey City, NJ USA; 3 Jaume I University, Castellón de la Plana, Spain

**Keywords:** Traditional gender beliefs, Intimate partner violence, Perceived stress, Depression, Rational emotive behavior therapy, MC-O’Kelly women’s beliefs scale

## Abstract

**Background:**

Research on traditional gender beliefs has highlighted their psychological impact and social implications for women. The purpose of this study was twofold. First, we aimed to adapt and validate the Spanish version of the Multicultural O’Kelly Women’s Beliefs Scale. Next, we explored its sources of validity evidence in relation to intimate partner violence, stress, and depression. Based on the Rational Emotive Behavior Therapy framework, traditional gender beliefs were expected to be associated with higher levels of intimate partner violence, stress and depression. We also expected to obtain a psychometrically-sound factor structure of the Multicultural O’Kelly Women’s Beliefs Scale.

**Methods:**

A sample of Spanish women (N = 322) completed the Multicultural O’Kelly Women’s Beliefs Scale, the Beck’s Depression Inventory II, the Modified Conflict Tactics Scale, and the Stress Perceived Scale. To test the psychometric properties of the Multicultural O’Kelly Women’s Beliefs Scale we implemented exploratory and confirmatory factor analyses and an analysis of the area under the curve.

**Results:**

Regarding the psychometric properties of the scale, statistical analysis revealed a one-factor dimensionality (Global traditionalism) and supported a reduction of items in the original instrument. The abbreviated version (eight items) obtained the best fit indices. Considering the association between traditional gender beliefs and psychological outcomes, we found that traditional gender beliefs were associated with increased severity of stress, depressive symptoms and reciprocal verbal aggression.

**Conclusion:**

The Spanish adaptation of the Multicultural O’Kelly Women’s Beliefs Scale provided a very short, psychometrically robust and clinically relevant measure of traditional gender beliefs. In addition to the association between traditional gender beliefs and mental health outcomes, an important finding was the relationship between traditional gender beliefs and intimate partner violence. Our scale might be used in clinical settings by helping women to identify their traditional gender beliefs and replace them by healthy and goal-oriented beliefs, which would also contribute in achieving a more egalitarian society.

**Supplementary Information:**

The online version contains supplementary material available at 10.1186/s12905-021-01572-2.

## Background

Wolfe's pioneering work on women's gender beliefs under the Rational Emotive Behavior Therapy (REBT) framework provided a women-centered perspective of traditional gender roles in clinical settings [83]. Based on Ellis's and Wolfe's research [24], O'Kelly [59] specified that, when these beliefs are transmitted from one generation to another, we can speak of traditional gender beliefs (TGBs). Gender roles would be the expression of TGBs found in rigid gender stereotypes. Wolfe et al. [86] defined TGBs as the set of beliefs (gender stereotypes) transmitted through a cultural learning process by which men and women think, feel, act and relate in a certain way (gender roles). Therefore, TGBs involve the upholding, both individually and collectively, of specific biased expectations regarding the behavior, preferences, responsibilities, activities, appearance, sensitivities etc. of individuals, often leading to the discrimination of those who do not follow these widely accepted stereotypes [90]. A paradigmatic example of TGBs is the biased acceptance of characteristics considered inherent to women (e.g., nurturing and caring) and men (e.g., self-reliance and assertiveness) [47]. Recent studies on biased beliefs highlight their considerable impacts on welfare [8] and their social implications (e.g., discrimination) for both women and minorities [28].

According to the Office of the High Commissioner for Human Rights [62], a gender stereotype "is a generalized view or preconception about attributes or characteristics that are or ought to be possessed by women and men or the roles that are or should be performed by men and women". In this sense, womanliness and manliness refer to traits, physical attributes, and behaviors that are based on the acceptance of the appropriate roles socially attributed to women or men. This generalized view consists of a shared belief system within the heart of a culture whereby specific characteristics are ascribed to individuals based on their sex [55].

Regarding gender issues, research around the world coincides in considering TGBs as essential features in relation to a range of individual beliefs, attitudes and decisions associated with gender: gender and health behaviors [6], gender and political participation [15], gender and occupation [21], gender and value beliefs [27], gender and intimate partner violence (IPV) [14, 29, 37], gender and cognitive abilities [31], gender and work-life preferences [45], gender and sexual orientation [54], and gender and feminist identity [78]. Research has also shown the influence of TGBs on women's psychological health, including depression [7, 16, 38] as well as stress and anxiety [3, 89]. In addition, TGBs have been associated with gender abuse [26, 51]. Therefore, the modification of TGBs might provide resilience resources to women and minorities [33].

Extensive literature has documented that beliefs about ourselves and others form the bedrock of many psychological issues that could be effectively addressed through psychological intervention [88]. However, before TGBs can be effectively targeted, it is essential to have psychometrically appropriate instruments to measure them. A measure of TGBs that has received attention from the research is the O'Kelly Women’s Beliefs Scale (OWBS; [59]). The original OWBS included 92 irrational beliefs about women’s traditional gender role [59], which were then reduced to obtain a 30-item, multicultural and faster-to-administer version [43, 44]. In both cases, items were believed to follow the Ellisonian assumption [23] that beliefs can be grouped in either three content areas (love and sex relationships, self-sacrifice and victimhood, and work and career) or four cognitive processes (demandingness, awfulizing, low frustration tolerance, and negative self-rating). However, neither the three- nor the four-factor solutions obtained sufficient support in previously published literature [44, 59]. This is problematic because the use of artificial scales might lead to misdirected interpretations of the participant’s scores and affect treatment plans. Thus, efforts to obtain a psychometrically sound version of the OWBS, such as the present one, are needed.

Several authors have explored irrational beliefs in the Spanish population, either validating existing measures or developing new ones. However, only a few of them have incorporated the cognitive processes proposed by REBT (demandingness, awfulizing, low frustration tolerance, and negative self-rating). Exceptions to this are the Attitudes and Beliefs Inventory (ABI; [9]), which has been adapted to the Spanish population [11], and a reduced version of the Irrational Beliefs Test (IBT, [35]), which was published by Calvete and Cardeñoso [12, 13]. More recently, the Scale of Irrational Contents and Styles–Basics was created to evaluate the content of irrational beliefs in relation to the types of inference in which they can be expressed (SICS-B, [68]). While useful for their administration in clinical samples, all these measures evaluate general irrational beliefs. Therefore, there is no instrument that can be used in Spanish-speaking countries to evaluate women's traditional gender beliefs from a REBT perspective. This is important because studies in Spain have shown that dysfunctional cognitive schemas are related to psychological symptons [49, 50]. In addition, studies have shown the importance of cognitive representations in the genesis and maintenance of gender-based violence [69], as well as in the predisposition to develop psychological disorders such as depression and stress in adolescents [1]. To manage both psychological concerns and gender-based violence, it is particularly useful to have empirically supported tools to assess the degree to which TGBs may be acting as a conditioning unconscious mediating factor (e.g., helping to explain why some women remain under abusive situations, or why others do not disclose their personal history of gender violence or do not ask for help).

The aim fo the present study was twofold. Firstly, we attempted to obtain a psychometrically sound factor solution of the MC-OWBS to be used in routine care for a cross-cultural detection of TGBs. Secondly, we investigated the hypothesis that there is an association between TGBs and depression, IPV, and perceived stress (sources of construct validity). Based on previous research, we expected a three- and four-factor solution based on content areas and processes to be difficult to replicate. We also expected that, once a robust factor structure was obtained, TGBs would be associated with more severe depressive symptoms, stress, and IPV. We hope that obtaining a psychometrically robust and valid measure of TGBs will help clinical practice and the prevention of mental distress and gender violence toward women.

## Methods

### Study design and participants

The sample comprised 322 adult Spanish women (M = 21.4, SD = 4.8). They voluntarily agreed to participate in the study and completed a series of questionnaires. Our sample size exceeded the generally accepted recommendation of five participants per parameter [39]. Regarding cohabitation status, 114 participants (36.8%) were living with their partner, and 196 (63.2%) reported having other cohabitation situations.

### Data collection

Participants were recruited using the snowball technique among the relatives and acquaintances of university students. Snowball sampling is a non-probabilistic sampling technique in which researchers begin with a small population of known individuals and expand the sample by asking initial participants to identify others who would be willing to participate in the study. The data collection was performed by REBT expert psychologists who contacted women university students. The initially contacted participants provided researches with contact information for women who might be interested in participating. All participants were notified with the identification and contact information of the researchers. The inclusion criteria included that the participants were adult women born or living in Spain for at least 10 years. Regarding the questionnaires, only the fully completed ones were included and the only exclusion criterion was to refuse to give consent to participate. Self-report paper-and-pencil questionnaires were used to collect the data. Participants’ consent was obtained by filling a data sheet containing personal information without any identification. This was done to maintain confidentiality and anonymity. The questionnaires took approximately 7–10 min to complete. Participants were informed by the researchers that they could leave the study at any moment. The current research complied with APA ethical standards for human research and was approved by the Research Ethics Committee of the University of Deusto (reference number: ETK-21/18-19).

### Measures

#### Traditional gender beliefs

To determine the participants’ adherence to traditional gender beliefs, we used the 30 irrational items from the multicultural version of the O’Kelly Women’s Beliefs Scale (MC-OWBS; [43, 44]). The MC-OWBS (see Additional file 1) is a Likert-type scale that indicates the degree of adherence to a series of TGBs (1 = *completely disagree*, 2 = *disagree*, 3 = *neither agree or disagree*, 4 = *agree*, and 5 = *completely agree*). The Colombian, multicultural version of the OWBS [43, 44] was adapted to the Spanish culture, rewording original local expressions. To ensure the comparability of measures, two bilingual REBT-certified supervisors checked whether the translated items matched the original. Gender discriminating terms were avoided. For example, “husband/male partner” was replaced by “partner.”

#### Perceived stress

Stress was measured using the perceived stress scale (PSS) originally developed by Cohen et al. [17]. Its Spanish version was validated by Remor and Carrobles [66]. This 14-item scale explores the degree to which people perceive daily situations as stressful (psychological stress). It is currently one of the most widely used scales to measure perceived stress and has obtained excellent psychometric properties in non-clinical samples [65, 77]. Participants have to report whether they can control some situations or whether they feel overwhelmed by them [63]. Response scales range from zero (*never*) to four (*very often*). The total score is obtained by reversing the scores of items 4, 5, 6, 7, 9, 10 and 13 and then summing all items. Higher scores indicate higher perceived stress levels. The reliability of the stress scale in the present study was excellent (Cronbach’s alpha = 0.85).

#### Depressive symptoms

To measure depression, we used the Spanish version of the Beck Depression Inventory-II (BDI-II; [70]). The scale is a 21-item multiple-choice questionnaire in which each question has four alternative answers (except items 16 and 18, which have 7 response options). The total score ranges from 0 to 63. According to the original BDI-II manual [5], categorical depression levels are: minimal (0–13), mild (14–19), moderate (20–28), and severe (29–63). The internal consistency of the BDI-II in the current sample was excellent (Cronbach’s alpha = 0.93).

#### Intimate partner violence

To evaluate IPV, we used the modified version of the Conflict Tactics Scale (M-CTS; [58]) derived from the Conflict Tactics Scale (CTS) created by Straus [74]. This scale measures whether a couple uses reasoning or aggression as tactics to resolve their conflicts. The M-CTS consists of 18 bidirectional items about the committed (18 items) or suffered aggressions (18 items) within the couple [58, 75]. It was validated in Spain by Muñoz-Rivas et al. [56], who identified four conflict resolution factors or tactics: (1) reasoning as a non-aggressive way of resolution (items 1, 2, and 3); (2) psychological or verbal aggression in the form of arguments, insults, or threats (items 4, 5, 6, 7 and 8); (3) average physical aggression, which includes harm or minor injuries (items 9, 10, 11, 12, 13, 14 and 15); and (4) serious physical aggression, which involves severe injuries (items 16, 17 and 18). The Likert response format indicates the frequency with which the aggressions occur: 1 (*never*), 2 (*rarely*), 3 (*sometimes*), 4 (*often*) and 5 (*very often*). Research has indicated poor internal consistency of the reasoning scale both as perpetrator and victim (α < 0.32) and acceptable reliability estimates (0.63 ≤ α ≤ 0.82) for the remaining scales [56]. In the present study, the reliability of the reasoning scale both as perpetrator and victim was also poor (0.28 ≤ α ≤ 0.34). The remaining internal consistency estimates were adequate (0.68 ≤ α ≤ 0.95). According to these internal consistency estimates obtained in the present and previous research, there is insufficient evidence to support the reasoning scale. Therefore, this scale was ignored in the analyses.

### Data analysis

Several statistical procedures were implemented to obtain a robust factor structure, including exploratory and confirmatory factor analyses (EFA and CFA, respectively) and an analysis of the area under the curve (AUC). Moreover, following past research [40], only a reduced number of items would be discriminative of depression (AUC analysis), which would support a reduction of items in the scale. The factor structures of the reduced version and the original 30-item versions were compared in an exploratory manner.

Previous research has supported a three-dimensional distribution of items in the multicultural version of the OWBS based on content areas, namely love, work, and self [43]. However, the psychometric properties of this three-factor structure have been questionable. In this study, three approaches were followed to obtain a robust factor structure for the OWBS: a confirmatory factor analysis (CFA), an exploratory factor analysis (EFA), and an item reduction procedure (AUC analysis and selection of discriminative items of depressive symptomatology).

The CFA was used to test the fit of the aforementioned three-dimensional structure based on areas. Because the OWBS has also been conceptualized to evaluate the four irrational processes of thinking proposed in REBT (i.e., demanding, awfulizing, frustration intolerance, and self-downing), a 4-factor solution based on these processes was also investigated in a CFA.

CFA is often preferred to an EFA because the latter is data-driven as opposed to theory-driven. Moreover, as indicated in past research, exploratory solutions often resulted in factor structures that are difficult to interpret due to cross-loadings and are difficult to replicate [67]. However, EFA is useful when the structure of a scale is unclear. Because this is the case for the multicultural version of the OWBS, an EFA was conducted to explore whether factor solutions and item distributions other than the ones tested in the CFA could obtain a better fit. One- to five-factor solutions were investigated. Solutions with more factors, which are less parsimonious, would be tested only if an adequate fit was not obtained with 1-to-5 factor solutions.

Finally, a reduction in the number of items (AUC analysis) followed by an EFA of the reduced set of items was used to obtain a more reliable factor solution for the scale. This strategy has been shown to be useful when attempting to obtain robust versions of a questionnaire and has additional advantages such as reducing the time required to complete the questionnaire [52]. To minimize predictability loss due to item elimination, an AUC analysis was carried out to select the items to be included in a shortened version of the MC-OWBS [40]. Depressive symptomatology was the criterion used in the AUC analysis. The cut-off to determine the existence of clinically relevant depressive symptomatology was 14, in line with past research [5].

Following these analyses, we explored sources of criterion validity with the version of the scale that obtained the best fit. The criteria used in this analysis of sources of validity were depressive symptoms, perceived stress, and IPV. We also explored demographic differences in traditional gender roles. In particular, an independent samples Student *t*-test was conducted to investigate whether differences in beliefs emerged as a function of their cohabitation status (e.g., whether or not cohabitating with a romantic partner was associated with differences in the participants’ beliefs about traditional gender roles). We did not explore differences in beliefs as a function of age and educational level due to the reduced variability in these sample characteristics.

The estimator used in the CFA and the EFA was the diagonally weighted least squares. This is one of the preferred options with ordinal observed variables [46]. Model fit was evaluated with the Chi-square test, the Tucker–Lewis index (TLI), the comparative fit index (CFI), and the root mean square error of approximation (RMSEA). TLI and CFI estimates over 0.95 and RMSEA scores below 0.05 indicate an excellent fit of the model to the data [32].

The AUC and the descriptive analyses were computed using IBM SPSS Statistics, version 22.0 [34]. The CFA and the EFA were conducted with Mplus, version 6.12 [57].

## Results

### Results of the CFA, EFA, and AUC

As seen in Table 1, the 3-factor CFA obtained poor fit indices, especially when observing the statistics comparing the proposed model against the null model (i.e., TLI and CFI). In the EFA, solutions with at least three factors yielded an excellent fit to the data. Item distribution of the 3- and 4-factor solutions are presented in Table 2. These models are preferred to the 5-factor solution for parsimony and because the OWBS was conceptualized to evaluate 3 content areas (i.e., love, self, and work) and 4 thinking processes (i.e., demanding, awfulizing, frustration intolerance, and self-downing).

**Table 1:** Results of the confirmatory and exploratory factor analyses of the multicultural version of the O’Kelly Women’s Beliefs Scale

Factors	Chi-square	*p*	TLI	CFI	RMSEA (90% CI)
3-CFA	1123.09	< .001	0.879	0.888	0.075 (0.069, 0.080)
4-CFA	1109.39	< .001	0.650	0.679	0.074 (0.069, 0.080)
1-EFA	1247.30	< .001	0.860	0.870	0.080 (0.075, 0.085)
2-EFA	802.63	< .001	0.924	0.934	0.059 (0.054, 0.065)
3-EFA	616.43	< .001	0.948	0.958	0.049 (0.043, 0.055)
4-EFA	508.51	< .001	0.961	0.971	0.043 (0.035, 0.049)
5-EFA	443.81	< .001	0.966	0.977	0.040 (0.032, 0.047)

CFA, confirmatory factor analysis; EFA, exploratory factor analysis; TLI, Tucker–Lewis index; CFI, comparative fit index; RMSEA, root mean square error of approximation

**Table 2:** Expected item distribution in the CFA and results of the EFA and AUC

Item	Expected CFA		EFA							AUC		
	Content	Process	Content			Process				AUC	*p*	95% IC
			F1	F2	F3	F1	F2	F3	F4			
1	Love	LFT	.67			.68				.53	.371	.46, .61
2	Love	LFT	.43		.44					.55	.151	.48, .63
3	Work	Awful								.53	.394	.46, .61
4	Self	Downing			.54			.47		.53	.487	.45, .60
5	Self	Downing	.41			.44				.57	.067	.49, .65
6	Self	LFT			.47				.60	.51	.846	.44, .58
7	Love	Demand	.74			.73				.55	.163	.48, .63
8	Work	Awful		.66			.66			.51	.887	.43, .58
**9**	Love	Awful			.78			.87		.61	.005	.53, .68
10	Self	Demand			.44				.55	.50	.894	.42, .57
11	Love	LFT			.83			.82		.57	.087	.49, .64
12	Work	Downing								.61	.004	.54, .68
13	Self	Awful								.56	.087	.48, .63
14	Love	LFT		.50			.45			.58	.033	.51, .65
15	Self	Demand			.60			.51		.58	.034	.50, .66
16	Love	Downing		.54			.49			.59	.015	.52, .67
17	Self	Downing		.50			.46			.63	< .001	.56, .71
18	Love	Awful	.43	.51		.45	.45			.60	.006	.53, .68
19	Work	LFT		.75			.73			.51	.901	.43, .58
20	Self	Downing		.73			.70			.55	.207	.47, .62
21	Work	Awful		.79			.80			.54	.284	.47, .62
22	Work	Downing		.84			.82			.52	.531	.45, .60
23	Self	Downing			.42				.55	.52	.519	.45, .60
24	Love	Demand			.87			.80		.55	.186	.48, .63
25	Love	Demand			.53					.60	.009	.53, .67
26	Love	Downing		.65			.61			.55	.205	.47, .62
27	Self	Downing		.61			.60			.55	.164	.48, .63
28	Self	Downing		.54			.55		.44	.54	.329	.46, .61
29	Work	Demand		.88			.86			.53	.432	.45, .61
30	Work	LFT		.60			.56			.57	.067	.49, .64

Expected CFA, expected item distribution in the confirmatory factor analysis; EFA, exploratory factor analysis; AUC, area under the curve; LFT, low frustration tolerance

Table 2 shows the results of the EFA (3- and 4-factor solutions) and the AUC. It also shows the distribution items according to the CFA (3 areas and 4 processes), which is used to compare the item distribution in the CFA and the EFA. Only factor loadings equal to or greater than 0.40 are reported, as recommended in previous research [79].

As seen in Table 2, a number of issues emerged in the 3- and 4-factor solutions obtained in the EFA. For example, several items had cross-loadings (e.g., items 2 and 18 in the 3-factor solution and items 18 and 28 in the 4-factor solution). In addition, a number of items had insufficiently meaningful loadings on both solutions (e.g., items 3, 12, and 13). Finally, the distribution of items largely differed from the assumed distribution (see CFA columns for a comparison).

Regarding the AUC analysis, as shown in Table 2, the items that obtained significant AUC estimates were items 9 (It is/it would be awful not to have a partner), 12 (I’m hopeless if I don’t help others at work to get on well together), 14 (It would be so uncomfortable if I challenged the decisions and advice of my partner that I could not stand it), 15 (I must have a child to be fulfilled), 16 (I am an unpleasant person if I challenge and do not accept the decisions and advice of my partner), 17 (If I put my desires or wishes first I am an unlikeable person), 18 (It would be awful if I did not satisfy the wishes of my partner) and 25 (I must have someone stronger on whom I can rely).

Due to the reduced number of items, an EFA with 1 and 2 factors was conducted with this 8-item version of the multicultural OWBS. Both solutions obtained excellent fit indices (1 factor: χ^2^ = 38.86, *p* = 0.012, TLI = 0.984, CFI = 0.986, RMSEA = 0.051, 90% CI RMSEA = 0.024, 0.077; 2 factors: χ^2^ = 12.29, *p* = 0.504, TLI = 1.000, CFI = 1.000, RMSEA = 0.001, 90% CI RMSEA = 0.001, 0.053). For parsimony reasons, the 1-factor solution was preferred. The internal fit of this 8-item reduced version of the multicultural OWBS was very good (Cronbach’s α = 0.79).

Considering the previous results, the reduced 8-item measure of the multicultural OWBS (MC-OWBS-RV) (see Additional file 2) was preferred over the original 30-item version. Reasons for this choice included poor fit of the confirmatory analyses with the expected item distribution according to past research and REBT principles [43, 44], as well as psychometric and conceptual issues with the exploratory solutions (e.g., items with cross-loadings or insignificant loadings and item distribution that is inconsistent with theoretical principles). This leads us to believe that respondents were not able to differentiate between the expected areas or processes when completing the questionnaire. By contrast, the 8-item version had excellent psychometric properties (model fit and reliability indices) and could be interpreted as representing a measure of general irrationality regarding traditional gender roles.

### Sources of criterion validity of the MC-OWBS-RV

As a final step towards obtaining a psychometrically and conceptually robust version of the multicultural OWBS, we tested sources of criterion validity of this reduced 8-item measure of the MC-OWBS-RV. The obtained correlations are shown in Table 3. Maladaptive or irrational beliefs, as measured with the MC-OWBS-RV, were associated with greater severity of stress (r = 0.28, *p* < 0.001; small size), depressive symptoms (r = 0.35, *p* < 0.001; medium size), and more verbal aggression both as perpetrator (r = 0.22, *p* < 0.001; small size) and as victim (r = 0.19, *p* < 0.001; small size). A milder but still significant association was found between irrational beliefs and average and serious physical aggression as perpetrator (r = 0.14, *p* = 0.011; small size), as well as between irrational beliefs and serious aggression as victim (r = 0.12, *p* = 0.039; small size).

**Table 3:** Means, standard deviations, and Pearson correlations between study variables

	Mean (SD)	Bivariate associations							
		2	3	4	5	6	7	8	9
1.MC-OWBS-RV	13.61 (4.21)	.28***	.35***	.22***	.19***	.14*	.06	.14*	.12*
2.Stress	2.84 (0.56)		.47***	.31***	.20***	.02	-.02	.03	.05
3.Depression	11.45 (3.56)			.19***	.13*	.09	.02	.12*	.11
4.Verbal-me	11.45 (3.56)				.84***	.31***	.29***	.03	.13*
5.Verbal-other	10.79 (3.36)					.36***	.33***	.11*	.12*
6.Average-me	7.52 (1.52)						.65***	.65***	.52***
7.Average-other	7.51 (1.78)							.27***	.58***
8.Severe-me	3.03 (0.46)								.68***
9.Severe-other	3.02 (0.17)								

MC-OWBS-RV, reduced version of the multicultural version of the O’Kelly Women’s Beliefs Scale; ***p < .001; *p < .05

### Demographic differences in the MC-OWBS-RV

The analyses indicated no differences in traditional gender role beliefs (t = 0.78, 95% CI = [− 0.57, 1.33], *p* = .435) when comparing women who cohabitated with their sentimental partner (mean = 13.85, SD = 4.43) and those who presented other cohabitation statuses (mean = 13.47, SD = 4.07).

## Discussion

The objectives of this study were two: (1) to obtain a transcultural useful instrument to measure TGBs with psychometric guarantees (good internal structure and adequate sources of criterion validity); and (2) to investigate the hypothesis that TGBs are associated with stress, depression and the use of maladaptive tactics to resolve conflicts (as a form of IPV). Using different methodological strategies, we obtained a reduced 8-item scale with very good psychometric properties, including criterion validity sources (depressive symptomatology, stress, and IPV). A one-factor solution, which was labeled as general irrationality, was parsimonious and had an excellent fit to the data.

Previous research on the original 92-item OWBS [59] and the 30-item MC-OWBS [43, 44] presented problems in their factor structure based on three areas (love, work, and self) or four cognitive processes (demanding, awfulizing, frustration intolerance, and self-downing). Two reasons may explain these results: (1) it is difficult for women to distinguish between areas and processes and/or (2) irrationality is an overall trait that transcends areas and processes and constitutes a personality feature. While this is difficult to ascertain at this stage, the preference for a general traditional beliefs factor, as proposed in the present study, is consistent even though the scale was originally developed to reflect three content areas and four thought processes [59]. Following a series of analytical strategies, exploratory and confirmatory analyses did not support an item distribution based on these three or four dimensions. Therefore, we propose a one-factor solution of the MC-OWBS-RV (i.e., global traditionalism), which offers a parsimonious factor structure that is moderately associated with important outcomes, namely depressed mood, stress, and IPV, in the expected direction.

Consistent with our proposed unidimensional structure of TGBs, the REBT’s idea of irrationality was initially viewed as a global concept [22]. It was only some years later that this conceptualization evolved to differentiate specific irrational beliefs in diverse areas and according to distinctive cognitive processes. This was further developed by Ellis and Wolfe [24] and extensively applied by Wolfe [80, 81] in allusion to women, covering conditions such as depression, relationship problems, work issues, sexual concerns, eating disorders, and gender abuse. Since Ellis and Wolfe's pioneering work, 48 inventories to assess rational and irrational beliefs have been developed, and many of them failed to report their factor analytic results according to the expected structures [19]. These outcomes may be explained by the use of EFA as opposed to CFA. However, the authors still concluded that there is no evidence to support the theoretical idea that the best way to conceptualize irrational or rational beliefs is through dividing items by processes or areas [19]. It has been suggested that perhaps all the processes, or a combination of some of them, represent a single latent variable [20]. Our results are in line with this alternative.

A strength of the present study is that the strategies used to obtain a psychometrically robust measure of TGBs were beyond the implementation of classic statistics (e.g., Cronbach’s alpha). We conducted different statistical approaches to investigate the psychometric properties of the instrument, combining modern analytical methods (e.g., CFA and AUC). This allowed us to obtain a new reduced version of the scale that meets a varied number of psychometric requirements. Psychometrically, the MC-OWBS-RV is an 8-item unidimensional measure with statements revolving around roles, insights, musts, and gender stereotypes that women might self-impose. The scale also reflects women’s insights into traditional cultural stereotypes involved in gender beliefs. These TGBs, as indicated in past research, diminish their roles as human beings, subordinating their values to what is socially accepted from a patriarchal society [60, 61].

Additional strengths of the MC-OWBS-RV include the fact that these beliefs are relevant cross-culturally [42, 44] and that it has a reduced number of items (8) with a good predictive competence. In relation to the latter, there have been some calls for routine outcome monitoring of patients during therapy [30], which should be performed more frequently (as opposed to the traditional episodic assessment in most randomized controlled trials). Having a reduced number of items, as in the MC-OWBS-RV, makes this more feasible. Ultimately, these eight gender beliefs may be used in goal-oriented clinical settings with the aim of alleviating symptomatology by helping women to become aware of and understand their thoughts and feelings, enhancing metacognitive capacities [64]. From a REBT framework, women and men acquire irrational beliefs from childhood onwards, but women might develop a self-concept affected by specific traditional gender beliefs acquired through social messages related to gender stereotypes that make them unable to minimize the disturbance over the hassles of life and promote the development of psychological disorders [84]. Recent studies have pointed to this important relationship between beliefs and clinical and sub-clinical problems [3, 10].

Irrational beliefs about traditional gender role may be addressed in clinical settings by helping women to identify them, dispute them, and replace them by rational beliefs. The therapeutic strategy is to identify unhealthy emotions and maladaptive behaviors, to investigate the irrational beliefs underlying them, and to learn how to restructure the dysfunctional cognitive processes. With psychotherapy, women can learn to replace an unhealthy emotion such as depression (which, for example, may be caused by low frustration tolerance) into a healthy emotion, such as sadness. In addition, they become aware that, although an adversity may trigger an unhealthy emotion, there is a chance to regulate their emotional response by developing a healthier mindset.

Regarding the relation between beliefs and mental health, our results are in accordance with previous research on the maladaptive role of irrational beliefs in depression and stress [3]. TGBs, as measured with the MC-OWBS-RV, relate to greater severity of stress and depression, which is of critical significance in clinical interventions. Psychologists are committed daily to the real need to help men and women overcome their mental problems, improve their relationships and change attitudes that prevent them from living healthily.

In addition to the association between TGBs and mental health outcomes, an important finding was the association found between TGBs and IPV. These findings are consistent with previous research on the relevance of TGBs and their consequences in IPV [76]. Past research showed that IPV could be understood as a bidirectional phenomenon in which both parties use violence and/or abusive behaviors against one another [41, 73]. This has significant implications for prevention purposes and underlines the critical importance of measuring TGBs. Because TGBs are relatively stable cognitive patterns, attitudes, and behaviors [82, 83], research shows that TGBs act in different contexts and over time [21]. Therefore, addressing TGBs would be crucial in achieving a more egalitarian society. Addressing women’s traditional gender beliefs might socially relevant. Specifically, it represents a real action aimed both at banishing unhealthy beliefs and at applying functional ways of thinking that improve women's ability to acquire new skills that enable them to fully develop in the society, especially in vulnerable groups such as battered women. Recent studies have shown that traditional gender beliefs justify, promote, and perpetuate violence against women [26] by holding irrational beliefs systematically transferred to relationships.

Regarding the prevalence of gender-based violence, it should be noted that the percentage of reported cases is systematically low. In Europe, only one third of abused women report partner abuse [25]. Irrational beliefs about traditional stereotypes may justify the subordination of women to men, tend to blame the victim, legitimize the abuser's behavior, and maintain myths about gender violence. The most common psychological consequences for victims are a loss of self-confidence, feelings of vulnerability, anxiety, and depression, but physical consequences or even death are also frequent. Official reports do not reveal the dimension of a problem that constitutes a dramatic reality, affects the life of many women, and violates some of their fundamental rights, such as dignity, justice and equality [4]. Encouragingly, there is empirical evidence about the effectiveness of cognitive re-education strategies in programs aimed at changing stereotyped beliefs in relation to the dependence and submission of women victims of partner violence [2]. Having a short, psychometrically-sound, and valid instrument might help in this direction by providing the Spanish-speaking professionals with a tool that helps them identify problematic beliefs, guide treatment, and monitor progress. We also expect to inspire other researchers around the globe to obtain similar cross-cultural adaptations.

Since REBT was created, 6 decades of subsequent research has fully evidenced the effectiveness of cognitive behavioral therapies [18]. Thus, REBT and other forms of cognitive-behavioral interventions are now widely recognized as empirically-supported psychosocial treatments [19]. As the cognitive approaches claim, core beliefs related to the self, the other, the world, or the future shape the way people think, feel and behave, by conditioning internal rules, attitudes, and assumptions derived from them [36]. Therefore, the early detection of maladaptive forms of thinking (e.g., TGBs) is extremely relevant in terms of prevention and treatment.

An important milestone in the field of public health is the elimination of gender violence. In this sense, the World Health Organization recommends cognitive behavior therapies as a way of alleviating the psychological consequences of the victims [87]. Therefore, we emphasize the need to identify and challenge irrational cognitions that may have devastating costs and consequences on individuals, communities, and societies, particularly women who suffer gender violence [14]. According to this objective, Zapata-Calvente et al. [91] highlighted that it is essential to change the beliefs promoted by gender socialization, “which foster male dominance and unequal relationships between men and women”. Consequently, assessing TGBs is necessary to both advance in gender research and to construct a fairer society. Again, having a reduced set of core TGBs in the MC-OWBS-RV might facilitate this task.

Beliefs have unique implications for understanding the impact of the experience of gender abuse [2]. When women think irrationally, they hold dysfunctional, inaccurate, unhelpful, judgmental and/or unrealistic beliefs that constitute a vulnerability factor for managing potential stressors. Gender violence is indeed a major public health problem in Spain [53] and globally [87]. Accordingly, the Spanish Government claimed in its Equality Delegation that there is fundamental work to be done toward changing our cultural model, attitudes, and values. To do so, traditional stereotypes need to be eliminated, and equality must be promoted. Similarly, freedom ought to be achieved, and all forms of violence against women need to be eradicated.

Unfortunately, many young women who suffered gender violence are unacknowledged victims who do not view their experience as a form of victimization [48]. Any phenomenon with a gendered view implies observing what is invisible [71] and requires questioning mental patterns such as TGBs that may have been established and naturalized over centuries [85].

As a final contribution, the present work investigated demographic differences in TGBs. Our analyses indicated comparable beliefs when analyzing women who cohabitated with their romantic partners and those with other cohabitation statuses. One might have hypothesized that women who cohabitate with their partner might be more exposed to TGBs, either due to some freedom loss inherent to cohabitating with a partner or due to imposed beliefs when the partner has a conservative view of masculinity/femininity. In our sample, however, these differences in TGBs according to partner cohabitation status did not emerge. It is possible that the characteristics of the sample, at least partly explain the findings. In particular, the sample included young and highly educated women and the literature has evidenced that TGBs are less frequent in this population [67]. Therefore, while cohabiting with a partner has been previously associated with more engrained TGBs [72], our results might indicate that the new generations are better prepared to face the challenges of cohabitating with a partner in terms of gender beliefs.

### Limitations

This study is limited due to the characteristics of the sample, which is restricted to mostly young women. However, this may be seen as an indicator of how education is changing—or not—the mindset of younger generations vis-à-vis traditional gender beliefs. Most of the participants were Caucasian and middle class, living in urban areas. Broader samples with women from different social and economic status, age, and geographic origins in the same country may reveal differences in the level of adherence to specific TGBs. The sexual orientation of the participants was unknown, which is another limitation of the study, and we cannot be certain whether the results indicate the presence of irrational thinking related to a specific sexual orientation. Even accounting for the limitations of the sample, it seems clear that there is a moderate association between TGBs, IPV, and their psychological consequences in terms of perceived stress and depression, which supports the utility of the proposed measure of TGBs.

## Conclusions

According to the findings, the conclusion of this study regarding the connection between women’s traditional gender beliefs and psychological outcomes, is that TGBs are associated with higher index of perceived stress and greater severity of depression. Considering the implications of TGBs for intimate partner violence, the results indicated a significant relationship with verbal and physical aggression. These findings contribute to a better understanding of the role that TGBs may have in women’s life. Wolfe (personal communication, August 12, 2020) states that “the most important application of REBT to women’s issues is identifying the should and must messages they get from their society starting with childhood; the goal is to feel better about oneself and the world, behaving in ways that lead to productive problem solving, improved communication and becoming more active in defining and achieving one’s goals in life”. Therefore, the present work represents an important step forward in this direction by providing a very short, psychometrically robust and clinically relevant measure of TGBs. Beliefs matter and can be changed. It is our hope that a cultural change regarding traditional gender beliefs may be promoted not only through policies but also through the daily work that psychologists carry out in therapeutic settings with the victims of cultural irrationalities. We hope that the existence of a short, 8-item measure of TGBs—designed from a multicultural perspective—that can be rapidly administered in routine care helps accomplish this goal.

## Supplementary Information

**Additional file 1.** Multicultural O’Kelly women’s beliefs scale. In this section the Multicultural O’Kelly women’s beliefs scale, in Spanish and English languages versions, can be consulted.

**Additional file 2.** Reduced version of the Multicultural O’Kelly women’s beliefs scale. In this section the Reduced version of the Multicultural O’Kelly women’s beliefs scale, in Spanish and English languages versions, can be consulted.

## Data Availability

The datasets used and/or analyzed during the current study are available from the corresponding author upon reasonable request.

## Abbreviations

AUC: Analysis of the area under the curve
BDI-II: Beck Depression Inventory-II
CFA: Confirmatory factor analysis
CFI: Comparative fit index
CTC: Conflict Tactics Scale
EFA: Exploratory factor analysis
IPV: Intimate partner violence
LFT: Low frustration tolerance
M-CTS: Modified version of the Conflict tactics scale
MC-OWBS: Multicultural O’Kelly women’s beliefs scale
MC-OWBS-RV: Reduced version of the Multicultural O’Kelly Women’s Beliefs Scale
MSSCI: Ministry of Health, Social Services and Equality
OHCHR: Office of the High Commissioner for Human Rights
OWBS: O’Kelly Women’s Beliefs Scale
PSS: Perceived Stress Scale
REBT: Rational emotive behavior therapy
RMSEA: Root mean square error of approximation
SICS-B: Scale of Irrational Contents and Styles-Basics
TGB: Traditional gender beliefs
TLI: Tucker–Lewis index
WHO: World Health Organization
