# Supplementary material for: The role of women's traditional gender beliefs in depression, intimate partner violence and stress: insights from a Spanish abbreviated multicultural measure
Source: BMC Womens Health. 2022 Jan 22;22:17. doi: 10.1186/s12905-021-01572-2 (PMC8783451; doi:10.1186/s12905-021-01572-2)
Supplement: Supplementary file 1 — Additional file 1. Multicultural O’Kelly women’s beliefs scale. In this section the Multicultural O’Kelly women’s beliefs scale, in Spanish and English languages versions, can be consulted. [file 12905_2021_1572_MOESM1_ESM.docx]

**MC–OWBS**

|  | **Spanish** | **English** |
| --- | --- | --- |
| 1 | Sería insoportable si no satisfago los deseos de otros, especialmente los de mi pareja. | If I did not satisfy the wishes of others, particularly my partner, it would be unbearable. |
| 2 | Podría ser insoportable si no tengo alguien fuerte en quien pueda confiar o apoyarme. | It would be unbearable if I did not have someone stronger than myself to rely on. |
| 3 | Podría ser un desastre si me tomo mi trabajo muy en serio. | It would be a disaster if I took my work too seriously. |
| 4 | No soy nada sin un hijo. | I am nothing without a child. |
| 5 | Actuar de tal forma que disguste a otros, muestra que soy una persona inútil. | If I act in such a way that I upset others it shows that I am a hopeless person. |
| 6 | Sería insoportable no responsabilizarme completamente de mis hijos(a)s. | If I did not take total responsibility for my child/children it would be unbearable. |
| 7 | Tengo que satisfacer los deseos de otros, en especial los de mi pareja. | I must satisfy the wishes of others particularly those of my partner. |
| 8 | Como mujer podría ser/es terrible tener una posición superior en el trabajo. | As a woman it would be/is terrible to have a senior position at work. |
| 9 | Sería o es terrible no tener pareja. | It is/it would be awful not to have a partner. |
| 10 | Debería cuidar de mis hijos(a)s todo el tiempo. | I should be responsible for the care of my children at all times. |
| 11 | Sería o es insoportable no tener pareja. | It would be/is unbearable not to have a partner. |
| 12 | Siento que no valgo si no ayudo a mis compañeros  de trabajo a congeniar. | I'm hopeless if I don't help others at work to get on well together. |
| 13 | Si no corro a ocuparme de las tareas de mi casa, podría ser una catástrofe. | If I did not run home to do the housework it would be a catastrophe |
| 14 | No podría soportar la incomodidad de cambiar las decisiones y consejos de mi pareja. | It would be so uncomfortable if I challenged the decisions and advice of my partner that I could not stand it. |
| 15 | Debo tener un hijo(a) para sentirme realizada. | I must have a child to be fulfilled. |
| 16 | Soy una persona desagradable si no acepto las decisiones y consejos de mi pareja. | I am an unpleasant person if I challenge and do not accept the decisions and advice of my partner. |
| 17 | Si antepongo mis deseos a los de otros, soy una persona antipática. | If I put my desires or wishes first I am an unlikeable person. |
| 18 | Sería terrible si no satisfago los deseos de mi pareja. | It would be awful if I did not satisfy the wishes of my partner. |
| 19 | No puedo soportar hacer cosas que como mujer son difíciles de entender (ej.: estudiar matemáticas) | I cannot stand doing things (e.g., mathematics) that are too difficult for me as a woman to understand. |
| 20 | Soy antipática si los compañeros de trabajo están disgustados o avergonzados porque sé mucho. | If others at work are embarrassed or upset by how much I know it goes to show that I'm an unlikeable person. |
| 21 | Tratar de tener mejor salario o condiciones de trabajo, podría ser terrible. | For me to try to get better pay/work conditions would be terrible. |
| 22 | Soy antipática si me esfuerzo por conseguir una posición de alta dirección en mi trabajo. | I am unlikeable person if I strive for senior position at work |
| 23 | Si no cuido todo el tiempo de mis hijos(a)s, soy una persona despreciable. | If I do not take total care of my children I am a worthless person. |
| 24 | Debo tener pareja. | I must have a partner. |
| 25 | Debo tener alguien fuerte en quien pueda confiar. | I must have someone stronger on whom I can rely |
| 26 | No podría ser una persona valiosa si actúo asertivamente con los hombres. | I would not be a worthwhile person if I acted assertively with men. |
| 27 | Soy una fracasada si me ocupo de mí antes que de los demás. | I'm a failure if I do not look after everyone else before myself. |
| 28 | Se reduce mi valía si no me ocupo de las tareas de la casa. | It reduces my self-worth if I do not take care of the home. |
| 29 | No debo tratar de hacer cosas que como mujer son difíciles de entender (ej.: estudiar matemáticas) | I must not try to do things (e.g., mathematics) that are too difficult for me as a woman to understand. |
| 30 | Sería tan desagradable tomarme mi trabajo muy seriamente, que no lo soportaría. | It is so unpleasant if I take my work too seriously that I could not stand it if I did. |
