# Supplementary material for: The role of women's traditional gender beliefs in depression, intimate partner violence and stress: insights from a Spanish abbreviated multicultural measure
Source: BMC Womens Health. 2022 Jan 22;22:17. doi: 10.1186/s12905-021-01572-2 (PMC8783451; doi:10.1186/s12905-021-01572-2)
Supplement: Supplementary file 2 — Additional file 2. Reduced version of the Multicultural O’Kelly women’s beliefs scale. In this section the Reduced version of the Multicultural O’Kelly women’s beliefs scale, in Spanish and English languages versions, can be consulted. [file 12905_2021_1572_MOESM2_ESM.docx]

**MC–OWBS–RV**

|  | **Spanish** | **English** |
| --- | --- | --- |
| 1 | Sería o es terrible no tener pareja | It is/it would be awful not to have a partner. |
| 2 | Siento que no valgo si no ayudo a mis compañeros de trabajo a congeniar | I'm hopeless if I don't help others at work to get on well together. |
| 3 | No podría soportar la incomodidad de cambiar las decisiones y consejos de mi pareja | It would be so uncomfortable if I challenged the decisions and advice of my partner that I could not stand it. |
| 4 | Debo tener un hijo(a) para sentirme realizada | I must have a child to be fulfilled. |
| 5 | Soy una persona desagradable si no acepto las  decisiones y consejos de mi pareja | I am an unpleasant person if I challenge and do not accept the decisions and advice of my partner. |
| 6 | Si antepongo mis deseos a los de otros, soy una  persona antipática | If I put my desires or wishes first I am an unlikeable person |
| 7 | Sería terrible si no satisfago los deseos de mi pareja | It would be awful if I did not satisfy the wishes of my partner. |
| 8 | Debo tener alguien fuerte en quien pueda confiar | I must have someone stronger on whom I can rely. |
